# Phytoremediation Using Bamboo to Reduce the Risk of Chromium Exposure from a Contaminated Tannery Site in Kenya

**DOI:** 10.5696/2156-9614-7.16.12

**Published:** 2017-12-18

**Authors:** Faridah. H. Were, Godfrey A. Wafula, Simon Wairungu

**Affiliations:** 1 Department of Chemistry, College of Biological and Physical Sciences, University of Nairobi, P.O. Box 30197-00100, Nairobi, Kenya; 2 Kenya Forestry Research Institute P.O. Box 20412-00200 Nairobi, Kenya

**Keywords:** phytoremediation, chromium exposure, tannery waste, bamboo species, land-based disposal

## Abstract

**Background.:**

This study examines an intervention strategy to reduce the risk of chromium (Cr) exposure. It follows a previous Cr exposure investigation, which revealed that large volumes of Cr-contaminated waste were burnt on site. The study site had a long history of land-based waste disposal since 1994.

**Objective.:**

The potential for phytoremediation using bamboo species to restore Cr-contaminated soil was evaluated.

**Methods.:**

Chromium levels and physico-chemical properties of the tannery and control soils were analyzed before transplanting six different bamboo species. Translocation, bio-concentration and bioaccumulation factors of the species were assessed for phytoremediation capabilities.

**Results.:**

Chromium levels in the tannery soils ranged from 1337.0 to 3398.0 mg/kg dw. The chromium levels were significantly higher (P < 0.05) than those of the control soils (0.20 to 2.34 mg/kg dw) and markedly exceeded the recommended limit of 100 mg/kg dw. The physicochemical properties of the tannery soils were also significantly varied (P < 0.05) compared to the control soils. In all cases, the species grown in the tannery soils were tolerant to a wide range of prevailing conditions. All of the bamboo species in the present study had a 100% survival rate in the tannery soils, except for D. birmanicus, which had a survival rate of 83.3%. Moreover, growth performance of the species in the tannery and control soils as evaluated by height and clump diameters did not vary significantly (P > 0.05). However, Cr levels in the tannery differed significantly (P < 0.05) among the species and rhizosphere soils. D. asper, B. vulgaris, D. membranaceus and B. blumeana had a bio-concentration factor (BCF) > 1 and translocation factor (TF) < 1, indicating that they are suitable for phytostablization. On the contrary, B. bambos had a bioaccumulation factor (BAF) < 1 and TF > 1, indicating potential for phytoextraction, while D. birmanicus showed no potential for phytoextraction or phytostabilization.

**Conclusions.:**

The present study identified D. asper, B. vulgaris, D. membranaceus and B. blumeana as suitable for restoration of Cr-contaminated tannery sites. Close monitoring of toxic metals is necessary during application of these species. Further studies are also recommended using a wide variety of bamboo species to optimize their application in phytoremediation.

## Introduction

Leather processing is one of the most important sources of chromium (Cr) exposure.^1–8^ This is because basic chromium sulfate is not fully utilized during the tanning process. Its uptake is estimated to vary between 55 and 70%.^1–4^ The residual amount usually escapes in aqueous effluent. Major exposure pathways include uncontrolled disposal of tannery wastes that result in degradation of the environment over a period of time.^1–4^ Land-based disposal of Cr-contaminated sludge from a poorly managed effluent treatment plant is the most significant source of exposure.^1–3^ For instance, in India, between 2000 and 3000 tons of Cr is discharged into the environment annually from tanneries.^1^,^4^ Indiscriminate release of Cr in soil and water resources is therefore a serious public health concern.^1–8^

Recent studies in tanneries by Were et al.^3^ found that huge volumes of waste are dumped in open fields. The dry solid wastes are burned, thereby contributing to elevated levels of airborne Cr within the tannery and its environs. Under those conditions there is a possibility of oxidation of trivalent chromium (Cr (III)) which is less harmful than the more toxic hexavalent chromium (Cr (VI)).^8^ Studies have further indicated that Cr in soil usually presents a combination of both Cr(III) and Cr(VI) as it undergoes a series of transformations such as oxidation, sorption, precipitation and dissolution.^7^,^8^ Oxidants such as dissolved oxygen and manganese dioxide (MnO2) are capable of converting Cr(III) to hexavalent Cr.^8^ Several investigations have detected considerable levels of toxic Cr(VI) in the surface water as well as groundwater.^1^,^4^,^8^ Moreover, at a higher pH value, Cr (VI) is more bio-available than Cr (III). It is in this form that Cr is highly oxidizing, soluble, and mobile and poses the greatest risk to human health and the environment.^3^,^4^,^8^ Hexavalent chromium is a powerful epithelial irritant and is also considered to be a human carcinogen.^1–8^

The common practice of dumping of Cr-contaminated waste in open fields and the subsequent discharge of Cr into water resources requires stricter controls.^1–8^ There are several techniques that have previously been applied as a strategy to clean up sites contaminated with heavy metals, including remediation by use of chemical and thermal methods.^6^,^9^ Most of these methods pose technical difficulties in attaining optimum results and require large financial investments.^6^ Furthermore, the usual methods of excavation and subsequent disposal of contaminated waste to dumpsites have the potential of spreading and shifting these contaminants to other locations.^1^,^3^,^9^

Phytoremediation is an economically feasible method that uses plants, which have exceptional metal-accumulating capabilities, to restore contaminated sites.^1^,^6^,^11^,^12^ The method is less disruptive to the environment and more acceptable to surrounding communities.^6^,^9^ There are a large number of plants that contribute towards heavy metal removal and demonstrate good potential for application in a remediation progam.^8^,^9^

**Abbreviations**
*ANOVA*: Analysis of variance
*BAF*: Bioaccumulation factor
*BCF*: Bioconcentration factor
*EC*: Electrical conductivity
*TF*: Translocation factor

These plants have unique and selective metal uptake abilities where they bio-accumulate, translocate or degrade contaminants.^6^,^9^,^11^,^12^ Bamboo species usually thrive in toxic environments with minimal maintenance and produce a large amount of biomass.^10–12^ They are usually harvested from two years onward for their diverse applications.^10–12^ In this study, six bamboo species were selected for their heavy metals accumulation and translocation potential to restore Cr-contaminated tannery soil. The establishment of bamboo species on the contaminated site is expected to stabilize the soil with extensive root systems and prevent wind and soil erosion. This will prevent the risk of spreading Cr to other areas; thereby reducing associated adverse health effects.^1–7^

This study is an intervention strategy to reduce the risk of Cr exposure, following a previous Cr exposure assessment, which revealed large volumes of Cr-contaminated tannery waste that was subsequently burnt in the open, contributing to elevated levels of Cr exposure.^3^ The tannery workers were found to have significantly reduced lung function, as well as respiratory and dermatological complaints associated with high levels of Cr exposure.^3^ The study is therefore part of comprehensive ongoing research activities aimed at reducing the risk of Cr exposure in tanneries.

## Methods

The study began in January 2015 and ended in January 2017.

### Site Description

The study area was a Cr-contaminated site within the selected tannery, on an acre of land in close proximity to residential areas.^3^ Its geographical coordinates are 1° 17′ 0″ South, 36° 42′ 0″ East. The site has had a long history of land-based disposal of waste from the tannery since 1994. On average, the annual consumption of chromium sulphate in the tannery is about ten tons.^3^ Chromium sulphate utilization during the tanning process was estimated to be at 60%, suggesting that over 4 tons of Cr are discharged on a yearly basis into aqueous effluent.^3^ Although the tannery has an effluent treatment plant, it is poorly managed, and the plant is located about 50 m away from the Nairobi River and adjacent to agricultural land.^3^ It is therefore a major potential source of Cr migration into soil and water resources.^1–7^ Table 1 presents a summary of activities within the tannery that contribute to Cr exposure.

**Table 1 —:** Description of Major Activities in the Tannery Associated with Chromium Contamination of the Tannery Site

**Activities**	**Sources of Chromium**	**Disposal Methods**
Loading and storing of chrome sulphate	Spillage of chrome within the storage area	Chrome spillage in powder form was swept and dumped in the open field
Weighing chrome	Spillage of chrome during weighing Residual amount of chrome in empty packaging materials	Chrome spillage in powder form was swept together with empty chrome packaging materials and dumped in the open field, and thereafter burned to reduce volume
Tanning process involved transferring, dosing and mixing chrome	Spillage of chrome in production areas Unutilized chrome (about 40%) is discharged as effluent during the tanning process The effluent treatment plant was poorly managed and resulted in sludge containing Cr	Cr-containing sludge was scooped from the treatment plant and dumped in the open field
Mechanical processes such as sammying, splitting, and trimming of tanned leather	Both solid waste and liquid containing Cr effluents were generated	Cr-containing sludge and tanned leather waste was dumped in the open
Shaving of leather	Shavings of the wet-blue pelt containing Cr were generated	The shavings were dumped and burned in the open field
Buffing of leather	Leather dust bearing Cr was generated	The fine leather dust was dumped in the open field

The general topography of the study area is flat, windy, and characterized by low rainfall with well-drained sandy-loamy soils suitable for irrigation of food crops, mainly vegetables and maize.^3^ There are several scattered boreholes in the area, contributing to the possibility of Cr leaching into the water. Furthermore, tannery washings are channeled to a nearby farm and the rest could drain into the Nairobi River.^3^ It is also plausible that Cr-containing leather dust and fumes are blown and settled as depositions.^3^ During rainy seasons, these depositions are then washed by surface runoff, spreading the contaminants further.^3^ Use of Cr-contaminated sludge as manure was further observed to be a common practice in the area, and could result in severe cases of contamination of soils, as well as water resources. There are several pathways through which Cr could migrate and accumulate along the food chain.^1–7^ The present study investigated the applicability of bamboo species in controlling the leaching and spreading of Cr from contaminated sites to surrounding areas.

### Sampling of Bamboo Rooted Cuttings

A total of eighty-four (N = 84) three-month-old rooted cuttings of six different bamboo species: Bambusa blumeana, Bambusa bambos, Bambusa vulgaris, Dendrocalamus asper, Dendrocalamus birmanicus and Dendrocalamus membranaceus were selected for phytoremediation. The rooted cuttings of 30 cm in height were nurtured in black polythene pots with Cr-free soil by the Kenya Forestry Research Institute. These species were selected on the basis of their homogeneity from a larger population by considering their shoot quality and adaptability to being established in the prevailing environmental conditions.^10^,^12^ Their potential for accumulating heavy metals in order to restore the contaminated sites was also evaluated.^11^,^12^

### Sampling of Soils and Transplanting of Bamboo Rooted Cuttings

The tannery site was secured prior to transplanting of the bamboo species. Seventy-two (N = 72) holes of approximately 60 cm in diameter and 30 cm in depth sufficient to accommodate the bamboo rooted cuttings, were dug at each of the sampling points.^11^ Holes were spaced 2 m apart in order for the bamboo species to cover the area that was under direct influence of land-based disposal of Cr contaminated waste. Following the same procedure, twelve (N = 12) holes were dug in a garden, which served as control samples. Rooted cuttings of each of the 6 selected bamboo species were then transplanted in a row and watered with an equal amount of Cr-free water. Each hole was re-filled with the same soil, which was also collected in triplicates in a clean polythene bag for determination of Cr level and physico-chemical properties. Each soil sample was considered as a representative of growing media for the corresponding bamboo species that was planted. The species were thereafter rain fed and maintained in a natural environment.

### Determination of Physico-chemical Properties of the Soil

Soil samples that were collected from the sampling points prior to transplanting bamboo species in the tannery and control sites were assessed for Cr content as well as physico-chemical properties. The properties included the pH, electrical conductivity (EC), moisture and organic matter content.^13^ The pH and EC were determined by vigorous mixing of 10 g of soil and 50 mL of water. A deionized water ratio (w/v) of 1:5 allowed soluble salts to dissolve and the ionic exchange to reach equilibrium. Then the pH and EC were determined using digital meters with a combination of a pH electrode and a 1-cm platinum conductivity cell. Moisture content was calculated by the mass difference before and after drying of soils samples at 105°C to a constant mass.^13^ The organic matter was expressed as a percentage (%) of carbon and calculated using the Walkley-Black potassium dichromate wet oxidation by the titration method.^14^ Particle size distributions were analyzed using dry sieving techniques.^13^

### Growth Performance of Bamboo Species and Sample Preparation

Height and clump diameter of the bamboo species were measured using a clinometer and diameter tape, respectively to evaluate growth performance after two years of transplanting.^10–12^ The clump diameter was determined by measuring the distance covered by a cluster or group of stems of bamboo growing from a common underground rhizome system. Subsequently, the whole bamboo plant and corresponding soil samples at 0 – 30 cm depth of rooting zone were carefully collected from each sampling point. Each bamboo plant was then separated into respective organs composed of roots and shoots (stems and leaves). They were then washed in water and rinsed thoroughly with deionized and distilled water in order to remove soil particles and debris.^15^,^16^ The plant materials were subsequently chopped into small pieces using a stainless-steel knife. The samples were dried at 70°C to a constant weight. They were then milled in a cyclone mill to a particle size of 0.3 mm.^9^,^15^ The samples were packed in zip lock polythene bags, and stored in desiccators prior to chemical digestion.

Similarly, soil samples were air-dried at 70°C until a constant weight was attained. Any clods and clumps were removed and samples were mixed homogeneously.^15^,^16^ The samples were then sieved through a 2-mm sieve to remove coarse particles prior to chemical digestion.

### Chemical Digestion of Plant Materials and Soil Samples

A sample of 0.5000 g of air-dried pulverized plant materials were digested using 7 mL of concentrated nitric acid (HNO3): 1 mL hydrochloric acid (HCI) (7:1) in a fluorocarbon polymer (PFA/TFM) closed system microwave. The vessel liner was equipped with an extraction fume system (Anton Paar, Australia). The microwave unit was equipped with a quartz power system (1400 W). After cooling the vessel, the clear liquid was diluted to 50 mL in acid-washed vials.

Dried ground soil samples of 1.5 g were transferred to the 100 mL digesting tubes. This was followed by addition of aqua regia, a mixture of 14 mL concentrated HNO_3_ and HCl, 70% (Fisher Scientific, UK) in a ratio of 1:3. The tubes were covered by a funnel and digestion at 140°C was carried out in a fume chamber using a digestion block (Gerhardt, Germany). This was heated until about 4 mL was left in the tube. The procedure was repeated by adding a further 14 mL of aqua regia and allowed to evaporate to a volume of about 4 mL. After cooling, the solution was filtered through Sartorius membrane filters (3 hw). The filtrate was then made up to a volume of 25 mL with de-ionized and distilled water prior to analysis of total Cr. Laboratory blanks were prepared in the same way by having all the components added during the digestion process without the corresponding plant materials or soil samples. All the digested samples including the laboratory blanks were then taken for the spectroscopic analysis to determine the levels of total Cr.

### Analysis of Total Chromium Levels

The plant materials, soil and blank samples were analyzed for levels of total Cr instead of Cr(VI) using inductively coupled plasma optical emission spectrometry (ICP-OES). This is due to the interconversion of Cr(VI) and (III) during sample preparation and chemical analysis, which poses an analytical challenge for the determination of Cr(IV) levels.^3.8^ Total Cr levels were therefore determined in the soils before transplanting bamboo species, and those of the corresponding rhizosphere soils, roots and shoots after two years of growth period. The levels were expressed in milligram per kilogram of dry weight (mg/kg dw) of the respective samples.

### Quality Assurance and Control

Samples were analyzed using adequate quality assurances and controls (QA/QC) to determine the reliability and accuracy of the results. Precautions were taken to avoid external contamination of the samples. All reagents used throughout the analytical procedure were of high purity analytical grade. Glassware was soaked in 0.5% (v/v) of HNO_3_ and rinsed several times with distilled and de-ionized water prior to use.

The levels of total Cr in the samples were determined at optimized operational conditions of the equipment. Reliability and accuracy of the method was ascertained by use of Standard Reference Materials, IPE106 and IPE108 for plant Cr and ISE952 and ISE955 for soil Cr. All Standard Reference Materials were obtained from Wageningen Evaluating Programs for Analytical Laboratories (WEPAL), Wageningen University, Netherlands. The actual values were obtained from the sample reading minus those of the blanks. Equipment drift was monitored through calibration verifications at an interval of every 10 samples analyzed. QA/QC was also established by inter-laboratory comparison of levels of Cr in 10 sets of randomly selected digested plant materials and soil samples analyzed at both the Mines and Geological Department, Analytical Laboratory Services, Kenya and Crop Nutrition Laboratory Services using ICP-OES. The correlation coefficient values (P = 0.05) were in the range of 0.950 – 0.980 for soil Cr and plant Cr (0.960 – 0.980), indicating reliable results. The range of linearity was determined by checking the linear regression coefficient (r^2^), which was acceptable when r^2^ > 0.995. The validity of the method was further ascertained by cross method checks and replication analysis. All samples were analyzed in triplicates and the average of the determinations was taken when the relative standard deviation was less than 5% to establish reliability of the results.

### Statistical Analysis

The samples were analyzed in triplicates and the data obtained was then reported as mean ± standard error (SE) using the Statistical Package for the Social Sciences (SPSS) program (version 17.0, SPSS Inc, Chicago, Illinois). Independent Student's t-test was used to compare parameters from the control and tannery site. Statistical analysis of variance (one-way and two-way analysis of variance (ANOVA)) at P < 0.05 was used to compare variables between and within the groups.

### Accumulation and Translocation of Chromium

The capability of six different bamboo species to take up Cr from tannery contaminated soils was evaluated by determining the bioconcentration factor (BCF), translocation factor (TF) and bioaccumulation factor (BAF). The BCF was defined as the ratio of Cr levels in the roots to that of the rhizosphere soil of the bamboo species, while TF was the ratio of levels of Cr in the shoots to that of the roots of bamboo species. The BAF was calculated as the ratio of levels of Cr in the shoots over that of the rhizosphere soil of the bamboo species. Bamboo species with a high BAF value (BAF > 1) are suitable for phytoextraction, while those with BCF (BCF > 1) and low TF (TF < 1) have the potential for accumulation of Cr in the roots, also known as phytostabilization. It should also be noted that a TF > 1 indicates that the species has the ability to translocate Cr from the roots to the aerial parts.^8^

## Results

### Chromium Content and Physico-chemical Properties of Soils

Table 2 presents the results of total chromium levels and physico-chemical properties of the tannery and control soils before transplanting six different bamboo species: B. blumeana, B. bambos, B. vulgaris, D. asper, D. birmanicus and D. membranaceus. The soil samples were representative of the growing medium for each of the bamboo species. Out of a total of seventy-two (N = 72) tannery soil sample results, two were excluded from this study: two of the twelve (2 of 12) D. birmanicus that failed to grow to maturity.

**Table 2 —:** Preliminary Total Chromium Levels and Physico-chemical Properties of Tannery and Control Soils

**Soil Parameters Prior to Transplanting Six Bamboo Species**								
**Parameter**	**Site**	**B. blumeana**	**B. bambos**	**B. vulgaris**	**D. asper**	**D. birmanicus**	**D. membranaceus**	**P-value <0.001**
**Cr levels in soil (mg/kg dw)**	Tannery (Mean ±SE)	2326.7±34.7	2300.3±119.6	2107.4±118.7	2332.4±127.5	2324.8±122.7	2187.3±122.1	0.627
	Range	2138.0 – 2542.0	1539.0 – 3234.0	1351.0 - 2439.0	1337.0 – 3398.0	1348.0 – 3308.0	1449.0 - 2981	
	Control (Mean ±SE)	1.69±0.46^b^	0.38±0.23^a^	0.44±0.11^a^	0.28±0.16^a^	0.50±0.05^a^	0.67±0.22^b^	0.043
	Range	1.23 – 2.15	0.15 – 0.61	0.33 - 0.54	0.12 – 0.43	0.45 – 0.54	0.45 - 0.89	
**P-value**		<0.001	<0.001	<0.001	<0.001	<0.001	<0.001	
**pH**	Tannery (Mean ± SE)	7.84±0.13	7.73±0.19	7.99±0.18	7.81±0.14	7.68±0.09	7.60±0.13	0.510
	Range	7.30 – 8.60	6.70 – 8.60	7.00 – 8.60	6.90 – 8.30	7.00 – 8.10	6.80 – 8.20	
	Control (Mean ± SE)	6.55±0.01^ab^	6.70±0.10^ab^	6.15±0.05^a^	6.95±0.05^ab^	7.00±0.00^b^	6.50±0.30^a^	0.027
	Range	6.50 - 6.60	6.60 – 6.80	6.10 – 6.20	6.90 – 7.00	7.00 – 7.00	6.20 - 6.80	
**P-value**		<0.001	0.552	0.002	0.032	0.014	0.125	
**EC (μS/m)**	Tannery (Mean ± SE)	173.3±5.2^ab^	177.8±4.2^b^	171.9±5.2^ab^	153.5±7.3^a^	150.8±6.3^a^	167.4±6.6^ab^	0.006
	Range	134.0 – 190.0	159.0 – 199.0	139.0 – 198.0	114.0 – 191.0	118.0 – 189.0	127.0 – 198.0	
	Control (Mean ± SE)	109.0±11.0	104.0±17.0	108.0±0.5	114.5±8.5	108.0±1.0	91.5±0.1	0.056
	Range	98.0 – 120.0	87.0 – 121.0	107.0 – 109.0	106.0 – 123.0	107 – 109.0	91.0 – 92.0	
**P-value**		<0.001	<0.001	<0.001	0.059	0.019	0.306	
**Moisture Content (%)**	Tannery (Mean ± SE)	31.4±3.1	34.5±3.1	35.6±2.5	32.3±1.6	33.1±2.0	33.1±2.4	0.861
	Range	20.1 – 52.9	20.9 - 50.2	18.9 – 48.7	22.8 – 38.4	22.9 – 45.3	22.6 – 50.2	
	Control (Mean ± SE)	31.8±0.9	29.7±4.1	30.8±0.4	24.5±1.1	27.8±1.0	22.7±0.3	0.059
	Range	30.9 – 32.6	25.6 – 33.7	30.5 – 31.0	23.4 – 25.6	26.8 – 28.8	22.4 22.9	
**P-value**		0.961	0.552	0.470	0.071	0.306	0.111	
**Carbon Content (%)**	Tannery (Mean ± SE)	3.36±0.09	3.18±0.06	3.43±0.05	3.34±0.08	3.49±0.11	3.40±0.09	0.148
	Range	2.9 – 4.0	2.8 – 3.4	3.1 – 3.7	2.8 – 3.6	2.6 – 4.1	2.5 - 3.9	
	Control (Mean ± SE)	2.00±0.10	2.45±0.05	2.05±0.05	2.05±0.25	2.05±0.15	2.05±0.25	0.468
	Range	1.9 – 2.1	2.4 – 2.5	2.0 – 2.1	1.8 – 2.3	1.9 – 2.2	1.8 - 2.3	
**P-value**		<0.001	<0.001	<0.001	<0.001	<0.001	<0.001	

Independent Student's t-test compares different parameters of the tannery and control soils; P-value <0.001 indicates a significant difference between the control and tannery parameters, and mean values followed by the same small letter within the same row do not differ significantly (One –Way ANOVA, SNK-test, α = 0.05)

Abbreviations: SE, standard error

The mean ± standard error (SE) of Cr levels of the tannery soils for the six bamboo species did not differ significantly (P > 0.05), although the levels of Cr in the tannery soils (N = 70) varied from 1337.0 – 3398.0 mg/kg dry weight (dw). In contrast, significantly higher (P < 0.05) mean levels of Cr in the soil of B. blumeana (1.69±0.46 mg/kg dw) and D. membranaceus (0.67±0.22 mg/kg dw) than those of B. bambos (0.38±0.23 mg/kg dw), B. vulgaris (0.44±0.11 mg/kg dw), D. asper (0.28±0.16 mg/kg dw) and D. birmanicus (0.50±0.05 mg/kg dw), ranging from 0.12 - 2.15 mg/kg dw were observed in the control soils. Nevertheless, Cr levels in the tannery soils for the respective bamboo species were significantly higher (P < 0.05) than those of the control soils and markedly exceeded the recommended limit of 100 mg/kg dw.^2^,^15^

With regard to physico-chemical parameters of the soils, the pH values for the tannery soils (N = 70) varied from slightly acidic to alkaline (6.70 – 8.60), but the mean values for the six bamboo species were not significantly different (P > 0.05). On the contrary, the pH values for the control soils ranged from slightly acidic to neutral (6.10 – 7.00). Furthermore, the tannery soils had significantly higher (P < 0.05) electrical conductivity (EC) than the control soils. The mean values of EC of the tannery soils for B. blumeana, B. vulgaris and B. bambos were significantly (P < 0.05) different. The soil corresponding to B. bambos had the highest EC value of 177.8±4.2 μS/m. On the other hand, the mean moisture content of the tannery and control soils for the bamboo species did not differ significantly (P > 0.05), whereas the mean carbon content in the tannery soils ranging from 2.5 – 4.1% was significantly higher (P < 0.05) than that of the control soils in the range of 1.8 – 2.5%.

Two-way ANOVA was applied to determine the influence of bamboo species and treatment type on the dependent variables (pH, EC, moisture content, carbon content and Cr levels) presented in Table 3. However, no significant interaction (P > 0.05) was found between bamboo species and treatment type.

**Table 3 —:** Influence of Treatment on Bamboo Species and Treatment Type on the Dependent Variables

**Factors (Dependent variables)**	**pH**	**Electrical Conductivity**	**Chromium Levels**	**Moisture**	**Carbon Content**
Bamboo Species	0.761	0.738	0.990	0.180	0.860
Treatment	<0.001	< 0.001	<0.001	0.037	0.002
Bamboo Species X Treatment Interaction	0.315	0.381	0.990	0.893	0.415

Two-Way ANOVA, P < 0.05 indicates a significant interaction between bamboo species and treatment type

### Growth Performance and Chromium Levels

Table 4 is a summary of the growth performance of six different bamboo species determined by height and clump diameter, and levels of Cr in the rhizosphere soils, roots and shoots after two years of growth. The average height and clump diameters between the species in the tannery and control sites had no significance differences (one-way ANOVA, P > 0.001). Furthermore, all the bamboo species showed a survival rate of 100% under the prevailing conditions of the tannery soils, except for D. birmanicus, where two of its twelve samples failed to grow to maturity.

**Table 4 —:**
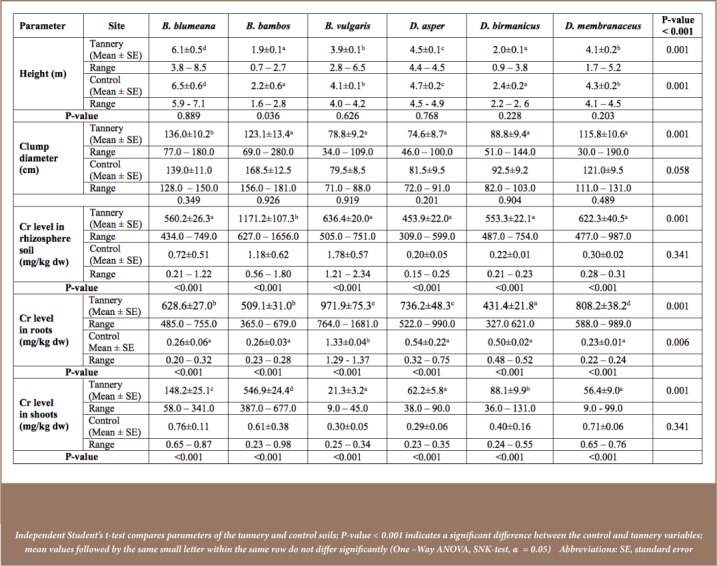
Summary of Growth Performance and Chromium Levels in Rhizosphere Soils, Roots and Shoots of Bamboo Species—Two Years of Growth

Significant (P < 0.05) variations in growth performance were observed among different species. For instance, in the tannery, the heights and clump diameters varied from 3.8 – 8.5 m and 77 – 180 cm for B. blumeana, 0.7 - 2.7 m and 69 – 280 cm for B. bambos, 2.8 – 6.5 m and 34 – 109 cm for B. vulgaris, 4.4 – 4.5 m and 46 – 100 cm for D. asper, 1.7 – 5.2 m and 30 – 190 cm for D. membranaceus, and 0.9 –3.8 m and 51 – 144 cm for D. birmanicus, respectively. The heights of B. bambos and D. birmanicus were significantly shorter (P < 0.05) than the rest of the species. However, the variations in the clump diameter for the species grown in the garden soil did not show significance differences (P > 0.05).

The results also showed a significant (P < 0.001 at 95% CL) reduction in the levels of Cr in the tannery soils (N = 70), where Cr levels varied from 1337.0 – 3398.0 mg/kg dw prior to transplanting the bamboo species and 309 – 1656 mg/kg dw after a two years growth period. The highest reduction was observed in the rhizosphere soils of D. asper, with Cr levels ranging from 309 – 599 mg/kg dw. However, the decrease was not significant (P > 0.05) among the species as evidenced by the mean values of Cr in the rhizosphere soils of B. blumeana (560.2±26.3 mg/kg dw), B. vulgaris (636.4±20.0 mg/kg dw), D. birmanicus (553.3±22.1 mg/kg dw and D. membranaceus (622.3±40.5 mg/kg dw) after two years. Interestingly, the soils of B. bambos in the tannery had significantly (P < 0.05) the least reduction in the levels of Cr after two years. Chromium levels of rhizophere soils were ranged from 627.0 – 1656 mg/kg dw, with a mean of 1171.2±107.3 mg/kg dw.

The mean levels of Cr accumulation in the roots and translocation in the shoots differed significantly (one-way ANOVA, P<0.001) among the species. The highest accumulation was observed in the roots of B. vulgaris compared to the other species grown in the tannery. The levels ranged from 764.0 – 1681.0 mg/kg dw with a mean of 971.9±75.3 mg/kg dw. In contrast, this species had the lowest translocation of Cr in the shoots, ranging from 9 – 45 mg/kg dw with a mean level of 21.3±3.2 mg/kg dw at the tannery site. This was followed by a significantly higher (P < 0.05) mean accumulation of Cr in the roots, in the descending order of: D. membranaceus (808.2±38.2 mg/kg dw), D. asper (736.2±48.3 mg/kg dw) and B. blumeana (628.6±27.0 mg/kg dw) as well as B. bambos (509.1±31.0 mg/kg dw). The lowest significant levels (P < 0.05) mean Cr level of 21.3±3.2 mg/kg dw, 56.4±9.0 mg/kg dw and 62.2±5.8 mg/kg dw were translocated in the aerial parts of B. vulgaris, D. membranaceus and D. asper, respectively, while the shoots of B. blumeana had a comparatively lower mean level of 148.2±25.1 mg/kg dw Cr.

B. bambos grown in the tannery soil significantly translocated (P < 0.05) the highest mean levels of 546.9±24.4 mg/kg dw Cr in the shoots and relatively lower mean level of 509.1 ±31.0 mg/kg dw Cr in the roots. The roots of D. birmanicus grown in the tannery had accumulated significantly the least (P < 0.05) mean level of 431.4±21.8 mg/kg dw Cr, while its shoots had comparatively lower levels of 88.1±9.9 mg/kg dw. Similarly, B. vulgaris grown in the control soils had the lowest level of Cr in the shoots, ranging from 0.25 – 0.34 mg/kg dw with a mean of 0.30±0.05 mg/kg dw. Bamboo species grown in the garden soils had generally low levels of Cr accumulated in the roots varying from 0.20 – 1.37 mg/kg dw. Nevertheless, B. vulgaris had the significantly highest levels (P < 0.05) of 1.33±0.04 mg/kg dw Cr, ranging from 1.29 – 1.37 mg/kg dw. Moreover, the levels of Cr translocated in the aerial parts of the bamboo species in the garden soils varied slightly from 0.23 – 0.98 mg/kg dw, although the differences in the levels were not significant (P > 0.05).

Table 5 presents the results of a two-way ANOVA on the effect of bamboo species and treatment type on the dependent variables. There was a significant (P < 0.05) influence between bamboo species and treatment type on levels of Cr in the rhizosphere soil, roots and shoots, while clump diameter did not show significant interaction (P > 0.05) between bamboo species and type of treatment.

**Table 5 —:** Effects of Bamboo Species and Treatment Type on the Dependent Variable—Two Years of Growth

**Dependent Variables**	**Height**	**Clump Diameter**	**Rhizosphere Soil Chromium**	**Root Chromium**	**Shoot Chromium**
**Bamboo Species**	< 0.001	< 0.001	0.003	0.012	< 0.001
**Treatment**	0.022	< 0.001	< 0.001	< 0.001	< 0.001
**Bamboo Species x Treatment Interaction**	0.613	0.650	0.003	0.012	< 0.001

Two-Way ANOVA, (P < 0.05) indicates significant interaction between bamboo species and treatment type.

Table 6 presents BCF, TF and BAF for all 6 species. The following bamboo species grown in the tannery soils: D. membranaceus, B. blumeana, D. asper and B. vulgaris exhibited a BCF>1 and TF<1, while B. bambos had a TF>1 and BCF<1 and D. birmanicus had a BCF<1 and TF<1. The control site had no particular trend as D. birmanicus and D. asper exhibited BCF>1 and TF<1. In D. membranaceus and B. blumeana, BCF>1 and TF>1 was observed. BCF<1 and TF>1 was exhibited by D. membranaceus, B blumeana and B. bambos. On the contrary, B. vulgaris had all parameters (BCF, TF and BAF) less than one.

**Table 6 —:** Accumulation and Translocation of Chromium in Six Different Bamboo Species in the Tannery and Control Site

**Site**	**Bamboo Species**	**Translocation Factor**	**Bioconcentration Factor**	**Bioaccumulation Factor**
**Tannery**	Bambusa blumeana	0.24±0.05	1.13±0.02	0.27±0.05
	Bambusa bambos	1.10±0.05	0.49±0.07	0.53±0.07
	Bambusa vulgaris	0.02±0.01	1.53±0.11	0.03±0.01
	Dendrocalamus asper	0.09±0.01	1.64±0.10	0.14±0.02
	Dendrocalamus birmanicus	0.21±0.03	0.79±0.05	0.16±0.02
	Dendrocalamus membranaceus	0.07±0.01	1.34±0.09	0.10±0.02
**Control**	Bambusa blumeana	3.19±1.16	0.84±0.68	1.90±1.19
	Bambusa bambos	2.54±1.72	0.31±0.19	0.48±0.07
	Bambusa vulgaris	0.22±0.03	0.83±0.24	0.18±0.03
	Dendrocalamus asper	0.80±0.13	2.57±0.43	1.47±0.07
	Dendrocalamus. birmanicus	0.16±0.08	2.21±0.03	1.83±0.79
	Dendrocalamus membranaceus	3.07±0.24	0.78±0.04	2.41±0.31

Abbreviations: SE, standard error

## Discussion

The present study found that levels of Cr in the soils were greatly varied and dumping of Cr-bearing waste from the tannery was a major source of Cr exposure. The levels of Cr in the tannery soils ranged from 1337.0 – 3398.0 mg/kg dry weight (dw) and were significantly higher (P < 0.05) than those of the garden soils (0.12 – 2.15 mg/kg dw). The former levels markedly exceeded the recommended limit of 100 mg/kg dw.^2^,^15^ This result is not surprising as the tannery site has been under the strong influence of land-based disposal of Cr-containing waste since 1994. The presence of higher levels of Cr in the tannery soils is an indication of the risk of exposure to humans as well as the environment.^1–8^,^15^,^16^ The residence time of Cr in soils is estimated to vary between 1000 and 10,000 years.^2^ The contaminated site in this case acts as a secondary pollution source for Cr, which is under the influence of several factors such as surface runoff, wind and soil erosion that is capable of spreading Cr contaminants to other areas. These findings are in agreement with those of Stępniewska and Bucior^15^ who reported significant levels of Cr contamination in soils, water and plants that were in close proximity to a tannery waste lagoon. As previously mentioned, most of the tanneries are located in ecologically fragile zones in the vicinities of residential areas, water sources and agricultural land.^1–3^

Quite a number of studies have revealed that toxicities of Cr are dependent on its speciation, and Cr(VI) is highly toxic, oxidizing, and more mobile and soluble than Cr (III).^3^,^4^,^8^,^16^ Similarly, levels of Cr that are bioavailable in soils are strongly linked to pH as a function of solubility and electrical conductivity among other physico-chemical properties of the soils.^8^ Trivalent Cr is easily converted to Cr(VI) when the pH value is greater than 6.0.^8^,^16^ In the present study, the pH values varied from 6.1 – 7.0 and 6.7 – 8.6 for the control and tannery site, respectively. The prevailing conditions of the soils therefore seemed to favor the transformation of Cr(III) to Cr(VI). However, the actual mechanism involved in accumulation and translocation of Cr in plants as well as the associated soil chemistry is complex given that Cr(III) is nonessential in plants.^8^ It should be emphasized that the availability of Cr(III) is a health risk due to possible conversion to Cr(VI). Symptoms of Cr(VI) toxicities in plants are diverse and inhibitions in growth as well as limited development of various organs are among the common indicators of toxicity.^8^ In the present study, the bamboo species had a survival rate of 100% for all the species grown except for D. birmanicus, where 16.7% of this species failed to reach maturity. Most species were therefore tolerant to a wide range of conditions, particularly those of the tannery site that had varied physico-chemical properties. Overall, the species were healthy and the average growth performance evaluated by height and clump diameter between those grown on the tannery and control soils did not show a significant difference (P > 0.05).

The present study also revealed a notable reduction of Cr levels from the tannery soils after a two year growth period for the bamboo species. High variabilities in the levels of Cr accumulated and translocated in various parts of the bamboo species as well as in their corresponding rhizosphere soils were also observed. The significantly high (P < 0.05) levels of Cr in the roots and shoots were therefore derivatives of Cr from the tannery soils. However, it is difficult to interpret these results and assess the actual amount of Cr that was bioavailable in the soils for uptake by the bamboo species.^8^,^16^ This is due to the complexity of soil chemistry that involves speciation of Cr and mechanisms for Cr uptake.^8^ Nonetheless, the differences in accumulation and translocation of Cr as well as growth performance among diverse bamboo species are very useful data for the selection of suitable species for restoration of Cr-contaminated tannery sites. There are more than 15 tanneries operating in Kenya and effluent treatment plants are poorly managed.^3^ Several studies have further demonstrated the success of using bamboo species for phytoremediation of heavy metals.^10–12^ This is consistent with numerous phytoremediation techniques recommended by the United States Environmental Protection Agency as remediation strategies for heavily contaminated sites.^21^,^22^

The mean level of Cr accumulated in roots of each of the species grown in the tannery was in the descending order of B. vulgaris, D. membranaceus, D. asper and B. blumeana. In contrast, roots of B. bambos and D. birmanicus accumulated lower levels of Cr. The variability could be attributable to different levels of Cr in the rhizosphere soils and genetic make-up among the species.^8^ The bioconcentration factor, translocation factor and bioaccumulation factor are important parameters in assessing the species' capabilities to tolerate, accumulate and transfer Cr to other parts of the species.^8^,^9^,^11^ In this study, D. asper, B. vulgaris, D. membranaceus and B. blumeana had a BCF > 1 and TF < 1, indicating potential for phytostabilization.^8^,^9^ On the contrary, B. bambos had a BAF < 1 and TF > 1, suggesting their potential for phytoextraction while D. birmanicus had neither potential for phytoextraction nor phytostabilization as the indices were all less than one. Only B. bambos seemed to show a real potential for phytoextraction with a Cr level of 546.9±24.4 mg/kg dw in the shoots. However, B. bambos cannot be classified as a hyperacummulator since it did not translocate more than 1000 mg/kg dw of Cr in the aerial part.^8^,^17^,^21^ In general, the bamboo species grown in the garden soils did not have a specific way or trend in accumulating or translocating Cr, perhaps due to low levels of Cr in the soil.

Our data suggest that Cr was mainly accumulated in four species with high retention in the roots of D. asper, B. vulgaris, D. membranaceus and B. blumeana, with less translocation in their aerial parts. The capability of sequestering high levels of Cr in the roots is a clear indication that the species are good candidates for phytostabilization of Cr.^8^,^9^ This perhaps explains the mechanism involved in assisting the species to tolerate elevated levels of Cr. Yoon also reported accumulation of heavy metals mainly in roots, and established the promising potential of these plants in excluding heavy metals.^9^ Similar results have been reported in several studies, where high levels of Cr were accumulated in roots with poor translocation in the shoots.^2^,^8^ This was noted as beneficial by ascertaining safe edible aerial parts of the species.^2^,^8^,^9^ The World Health Organization gave recommendations that Cr levels should not exceed 19 mg/kg in plants grown in unpolluted soils.^2^ However, plants grown in Cr-contaminated soils usually have levels that may increase significantly in the aerial parts.^16–17^ Earlier studies have also shown that Cr is accumulated in roots followed by stems and small amounts in the leaves.^8^,^15^

There were no significant differences between the average growth patterns of bamboo species grown in the tannery and control soils (t-test, P > 0.05). There were, however, significant variations (one-way ANOVA, P < 0.05) among the six different bamboo species, where B. bambos and D. birmanicus showed relatively slower growth than those of the other four species. The four species had dense rooting systems and a 100% survival rate, implying great tolerance to significant levels of Cr. Furthermore, a combination of extensive root systems and wider clump diameter stabilizes the soils, thereby reducing the amount of Cr entering into water sources through surface runoffs or migration to the ground water.^12^,^18^ This is an important factor that decreases mobility and bioavailability of Cr from the tannery soils to other areas. Previous studies are in agreement concerning the restriction of Cr in roots inhibiting its mobility into ground water, as well as bio-availability in the food chain.^18^,^20^ Accumulation and exclusion have been extensively discussed as critical strategies by which plant species may respond to elevated levels of Cr.^8^,^18^ This means that exclusion could be one of the tolerance strategies established by the four bamboo species, where translocation of Cr to the aerial parts is reduced, thereby minimizing the possibility of Cr entering into the food chain.^3^

**Figure 1 —:**
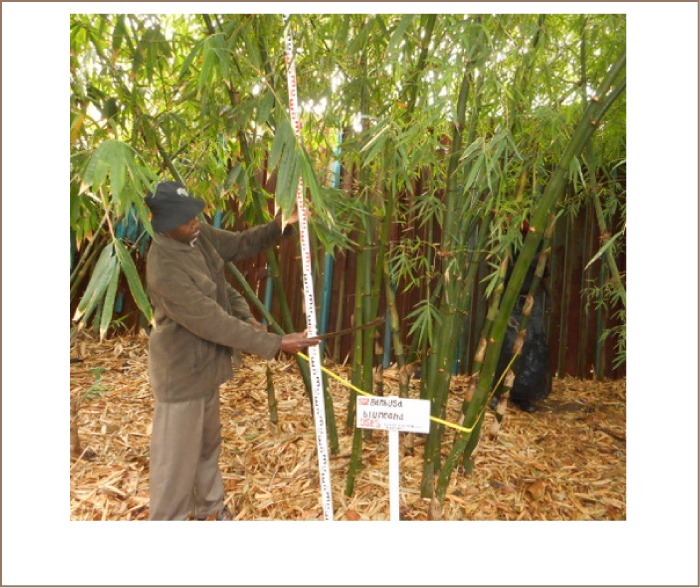
Evaluating growth of B. blumeana by determining the height using clinometer. This is after two years of growth during phytoremediation of a chromium contaminated site (Source: F. Were & Cutesy of KEFRI)

**Figure 2 —:**
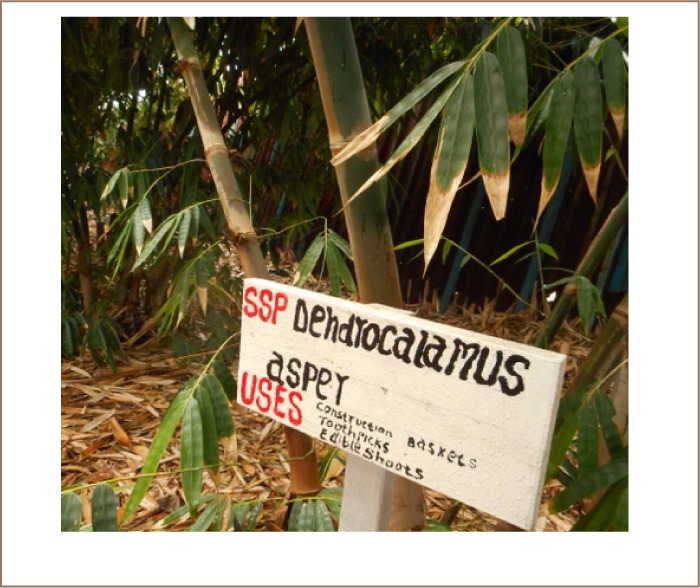
Asper after two years of growth during phytoremediation of a chromium contaminated site (Source: F. Were & Cutesy of KEFRI)

**Figure 3 —:**
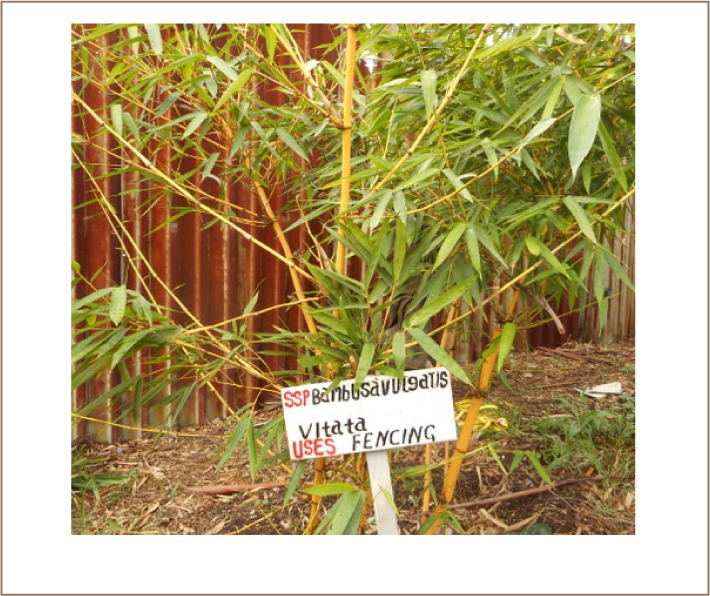
vulgaris after two years of growth during phytoremediation of a chromium contaminated site (Source: F. Were & Cutesy of KEFRI)

**Figure 4 —:**
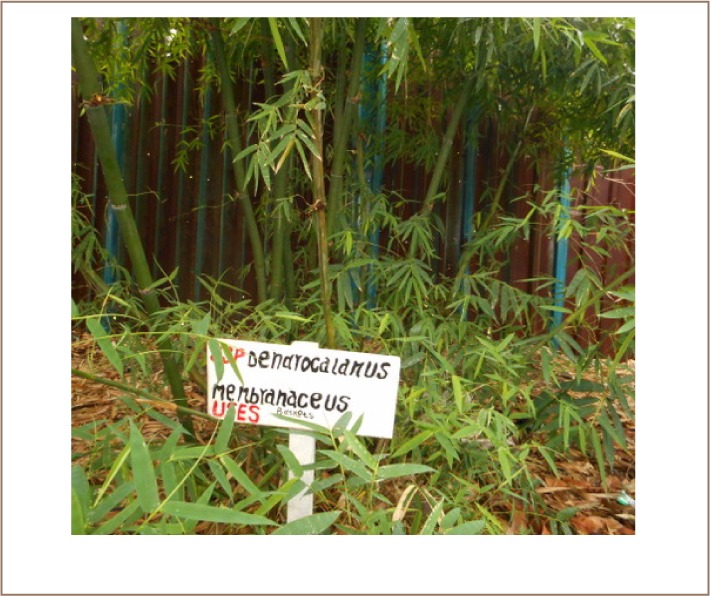
membranaceus after two years of growth during phytoremediation of Cr contaminated site (Source: F. Were & Cutesy of KEFRI)

Moreover, recent studies revealed open burning of Cr-containing waste, which poses great harm to the surrounding ecosystem.^3^ The hazardous residues are susceptible to wind erosion, with the possibility of being blown to adjacent areas, including the Nairobi River. The establishment of suitable bamboo species to mitigate the degraded tannery sites minimizes the spread of Cr to neighboring areas and reduces the risk of Cr exposure. Phytostabilization is therefore an important strategy that stabilizes Cr in a manner that prevents the associated risk of exposure.^8^,^18^ Studies have further revealed that this technique decreases Cr mobility and reduces the likelihood of Cr entering into the food chain.^2^,^8^,^18^ The four bamboo species are particularly fast growing with large biomass as observed in Figures 1 to 4. This prevents water seeping through the soil and inhibits soil and wind erosion, thereby preventing spread of Cr contaminants through surface runoff water that includes fugitive airborne Cr.^3^

It is worth noting that the four species are capable of attaining a height of 30 m after five years of growth.^10–11^ In particular, B. vulgaris grown in the tannery soils for two years had an average height of 3.9±0.1 m ranging from 2.8 – 6.5 m, and a relatively low mean level of 21.3±3.2 mg/kg dw Cr in the aerial parts. Moreover, the shoots of B. vulgaris are edible and may not pose a risk to animals. Soil removal due to leaf fall and disposal of biomass are also not necessary.^8^,^18^ The cost and degree of disruption is therefore minimal and at the same time the ecosystem restoration is enhanced.^9^ The species is effective in preventing migration of Cr from a highly contaminated site to fragile ecological zones.^11–12^ The main concern is however accumulation of Cr in the roots and possible release to the environment. Long-term maintenance of the species is therefore important for sustainability of the phytoremediation program. On the other hand, caution should also be taken to prevent the other species from transferring Cr into the food chain through translocation of elevated levels of Cr to the aerial parts. Further research on these species is highly recommended to optimize phytoremediation techniques in restoration of several contaminated sites as there are a number of native and exotic bamboo species in existence in Kenya.^10–12^

There are several limitations to the present study. In some cases, large amounts of fallen leaves, bearing Cr as shown in Figures 1 to 4 were not removed, which could have contributed to the Cr burden in the rhizosphere soils upon decomposition. Furthermore, total Cr was quantified in place of Cr(VI), as a result of analytical challenges associated with interconversion of Cr(III) and (VI). In addition, height and clump diameter were used in this study to estimate growth performance of bamboo species as an important factor in phytostabilization strategies, although determination of fresh and dry weight are regarded as the best biometric parameters in the determination of biomass.

## Conclusions

The study has clearly shown that D. asper, B. vulgaris, D. membranaceus and B. blumeana are remarkable species that can tolerate Cr-contaminated environments and are capable of excluding Cr from the soils. Bamboo clumps are versatile and have diverse applications. In all cases, close monitoring of toxic metals is necessary during utilization of these species, especially those grown in areas under the remediation program. B. vulgaris has emerged as the best species in mitigation of Cr-contaminated sites since it limits translocation of Cr in the aerial parts, and hence in the food chain. B. vulgaris is recommended for phytostabilization of Cr-contaminated sites as they prevent the mobility and migration of Cr to fragile ecological systems. They also have high levels of tolerance to physico-chemical stresses of soils and re-establish at sites where natural vegetation has failed to grow. Long-term monitoring strategies are critical to investigate related toxicities and ecological impact when used for phytoremediation since Cr remains in the roots after accumulation.
